# Methylation-regulated miR-124-1 suppresses tumorigenesis in hepatocellular carcinoma by targeting CASC3

**DOI:** 10.18632/oncotarget.8266

**Published:** 2016-03-22

**Authors:** Ling Xu, Weiqi Dai, JingJing Li, Lei He, Fan Wang, Yujing Xia, Kan Chen, Sainan Li, Tong Liu, Jie Lu, Yingqun Zhou, Yugang Wang, Chuanyong Guo

**Affiliations:** ^1^ Department of Gastroenterology, Shanghai Tenth People's Hospital, Tongji University School of Medicine, Shanghai 200072, China; ^2^ Department of Gastroenterology, Shanghai Tongren Hospital, Affiliated to Shanghai Jiao Tong University School of Medicine, Shanghai 200336, China

**Keywords:** miR-124-1, hepatocellular carcinoma, methylation, CASC3

## Abstract

This study was to investigate the roles and mechanisms of miR-124-1 in hepatocellular carcinoma (HCC). We analyzed the expression of miR-124-1 in human HCC tissues and cell lines. Luciferase reporter assays were used to analyze the target of miR-124-1. Human HCC cell lines were transduced with lentiviruses expressing miR-124-1, and proliferation and colony formation were analyzed. The growth of human HCC cells overexpressing miR-124-1 was assessed in nude mice. The expression of p38-MAPK, JNK, ERK and related signaling molecules was detected by western blotting and immunohistochemistry. Our results showed that miR-124-1 levels were reduced in HCC tissues and cell lines compared with those in adjacent non-cancer tissues and normal liver cell lines respectively. Downregulation of miR-124-1 in HCC cell lines were attributed to hypermethylation of its promoter region. Overexpression of miR-124-1 inhibited HCC cell proliferation *in vitro*, whereas miR-124-1 was correlated with clinicopathological parameters of HCC patients. HCC cell-mediated overexpression of miR-124-1 in nude mice suppressed tumor growth. Cancer susceptibility candidate 3 (CASC3) was identified as a direct target of miR-124-1 by computational analysis and experimental assays. MiR-124-1-mediated downregulation of CASC3 resulted in the inactivation of p38-MAPK, JNK and ERK. Our findings provide potential new targets for the prevention or treatment of HCC.

## INTRODUCTION

A large number of studies have shown that alterations in chromosome number and the structure of tumor cells play an important role in carcinogenesis. These changes have led to the identification of crucial oncogenes and tumor suppressors, which is essential for a better understanding of the pathogenesis of cancer and for clinical applications [1]. Chromosomal alterations including homozygous deletion and loss of heterozygosity are the most common form of genetic variation. Hepatocellular carcinoma (HCC), which is one of the most aggressive and prevalent cancers worldwide [2, 3], is a multi-phase, multi-factor, multi-gene and complex process. The development and progression of HCC is significantly correlated with the absence of more than 10 chromosomes, including loss of heterozygosity (LOH), and a high rate of genetic variation. Specific chromosome losses result in imbalance in a specific arm. If the chromosome region containing a tumor suppressor is lost, consistent with the loss of tumor suppressor genes contributing to tumorigenesis.

MicroRNAs (miRNAs) are a class of short endogenously expressed RNA molecules that regulate gene expression by binding directly to the 3′ untranslated regions (3′UTRs) of target mRNAs, either blocking their translation or causing target mRNA degradation [4, 5]. MiRNAs have the potential to regulate at least 20–30% of all human transcripts [6], and are involved in almost all processes associated with cancer such as carcinogenesis, proliferation, metastasis, angiogenesis, and apoptosis, acting as either oncogenes or tumor suppressor genes [7–9]. Previous studies have shown that cancer-associated genomic regions (CAGRs) contain miRNA genes. Approximately 52.7% of human miRNA genes are located in chromosome fragile sites, including 42% of miRNAs that are located exactly in minimal regions of LOH or minimal regions of amplification in a variety of tumors [10]. These findings suggest that miRNAs in CAGRs might be the focal points of future research as oncogene or tumor suppressor candidates.

In the present study, we analyzed the role of miR-124-1 in HCC. We found that miR-124-1 is downregulated by methylation-mediated gene silencing in HCC and its downregulation is significantly associated with clinicopathological factors in HCC patients. We identified CASC3 as a direct target of miR-124-1 and found that miR-124-1 modulates the activity of the p38-ERK-JNK pathway by regulating CASC3 expression, which may play a role in HCC tumorigenesis.

## RESULTS

### Aberrant microRNAs in chromosomes 8p23.3-21.3 of HCC

**Figure 1:**
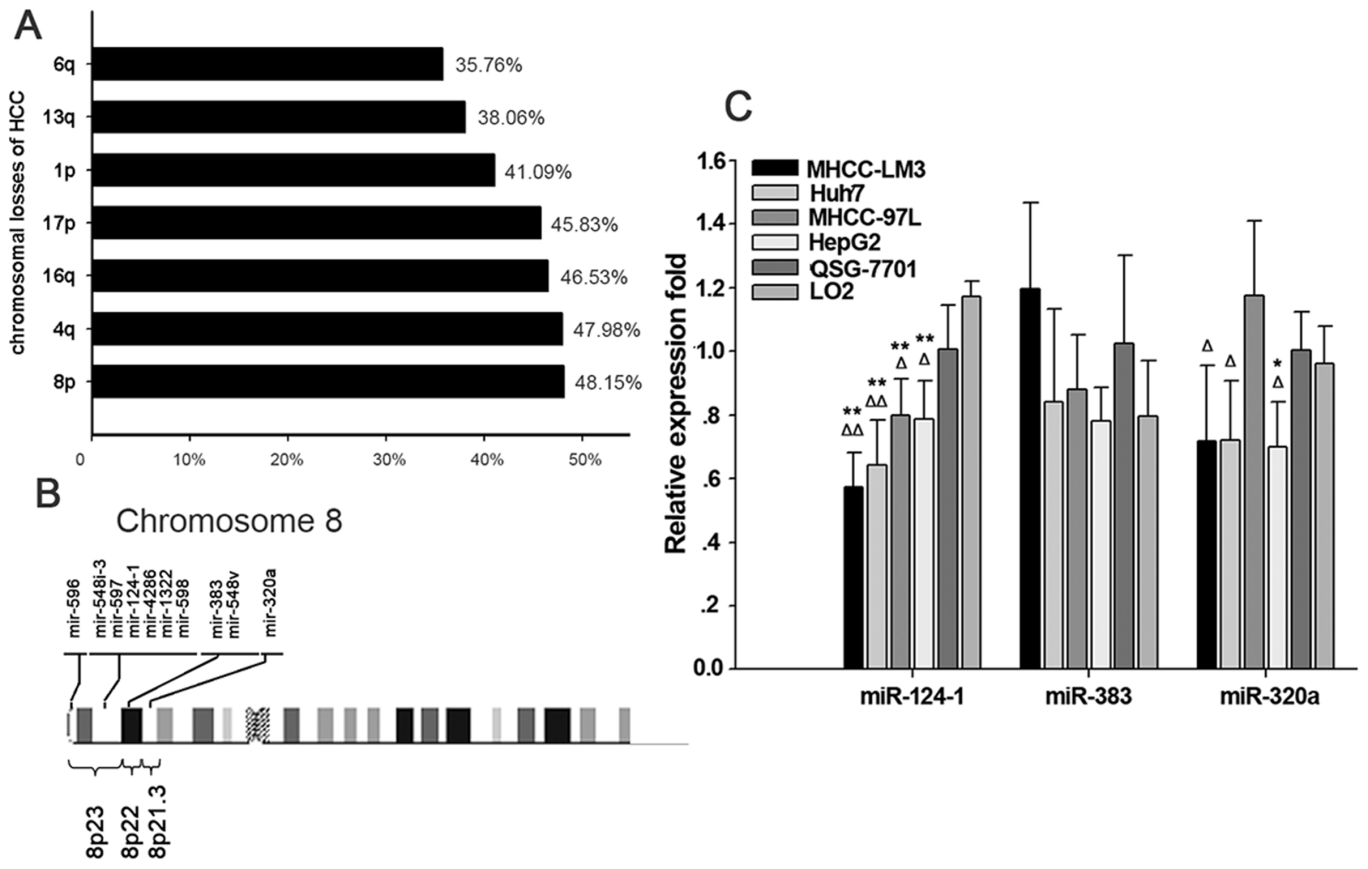
Aberrant microRNA expression in hepatocellular carcinoma **A.** Alignment of human microRNAs (miRNAs) with chromosomal instability sites in HCC. **B.** Altered breakpoint-associated miRNAs in chromosomes 8p23.3-21.3 of HCC. **C.** miRNA expression in chromosomes 8p23.3-21.3 in HCC cell lines. **P<0.01 vs. the normal human liver cell line LO2, *P<0.05 vs. LO2; ^ΔΔ^P<0.01 vs. normal human liver cell line QSY-7701, ^Δ^P<0.05 vs. QSY-7701.

The carcinogenesis of HCC involves an accumulation of genomic alterations. Analysis of chromosome gains and losses has led to the detection of oncogenes and tumor suppressors associated with the development of HCC. A high frequency of loss of chromosome 4q in HCC has been reported, suggesting that the dysfunction of specific tumor suppressor genes on this chromosome arm is involved in the development and progression of HCC. In the past two decades, many studies have focused on screening for chromosomal aberrations in HCC. To explore the most common chromosomal imbalances in HCC, we first searched for studies [15–40] analyzing genomic instability in HCC and extracted information on chromosomal losses associated with HCC. The results showed that the most common chromosomal losses in HCC are 8p (48.15%), 4q (47.98%) and 16q (46.53%) (Figure 1A, Supplementary Table 2 and Supplementary Table 3). This result is in agreement with the study by Guo et al., who found that copy number losses in 8p23.3-21.3 were present in > 55% of all HCC samples [3]. We then focused on losses of 8p23.3-21.3 and examined the candidate tumor suppressor miRNAs located within these regions (Figure 1B). We also explored their possible functions in hepato-carcinogenesis. The results showed that the expression levels of miR-124-1and miR-320a were downregulated in HCC cell lines with genomic deletion. Whereas miR-124-1 expression level was lower in HCC cell lines than those miRNAs expression in HCC cell lines. But the change of miR-383 expression was not statistically significant (Figure 1C). Some miRNAs were not detected in a variety of liver cancer cells such as miR-596, miR-548i-3, miR-597, miR-4286, miR-1322, miR-598 and miR-548v.

### Methylation mediated microRNA-124-1 expression in hepatocellular carcinoma cell lines

**Figure 2:**
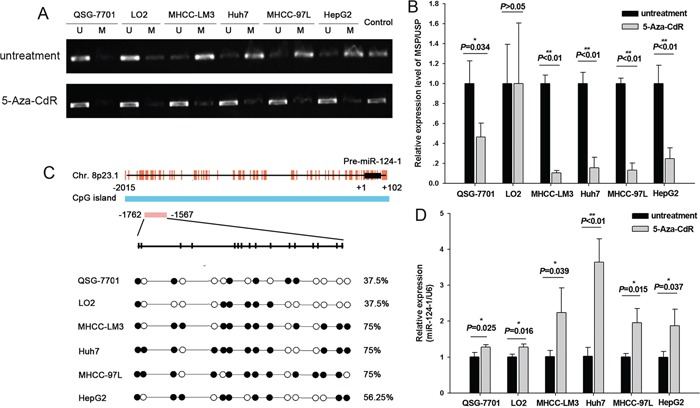
Methylation of miR-124-1 promoter in HCC cells **A.** PCR analysis in six cell lines, namely two normal liver lines (QSG-7701 and LO2) and four malignant lines (MHCC-LM3, Huh7, MHCC-97L and HepG2) before and after treatment with 50 mM 5-Aza-CdR. SssI methylase was the methylation-positive control. Results are shown as mean±sd (n¼3). **B.** MSP assay of the 5′ CpG islands of miR-124-1 in six cell lines. **C.** Bisulfite sequencing PCR analysis of miR-124-1 CpG island methylation in two normal and four malignant lines. The vertical bars denote individual CpG dinucleotides. The position of the pre-miR-124-1 sequence is indicated by black boxes, and the transcription start site is designated as +1. Each red vertical bar represents an individual CpG site, and the sequencing location is indicated by a red box. **D.** Relative expression levels of miR-124-1 are expressed as fold change relative to the untreated control. The assay was performed in triplicate wells and repeated three times, and similar results were obtained each time.

Based on previous studies showing that miR-124-1 methylation silences its expression in various human cancer cell lines, we investigated the effect of miR-124-1 methylation in HCC cell lines. In a previous study, we showed that miR-124-1 was expressed at higher levels in the normal liver cell lines QSG-7701 and LO2 than in the HCC cell lines MHCC-LM3, Huh7, MHCC-97L and HepG2. To confirm this hypothesis, we performed MSP for the CpG islands (CGI) of miR-124-1, which showed that miR-124-1 was hypermethylated in the four HCC cell lines compared to normal liver cells (Figures 2A and 2B).

Methylation of miR-124-1 promoter was further confirmed by analyzing its re-expression after treatment with a demethylating agent, 5-Aza-CdR, in HCC and normal liver cell lines. We found that the expression of miR-124-1 was restored in correlation with the reversal of methylation (Figure 2A, 2B and 2D). This further suggested that miR-124-1 is silenced by methylation in HCC cells.

Next, we examined the methylation status of miR-124-1 in two normal liver cell lines (QSG-7701, LO2) and HCC cell lines (MHCC-LM3, Huh7, MHCC-97L and HepG2) using bisulfite sequencing PCR. The DNA methylation pattern of miR-124-1 is shown in Figure 2C. It was found that the percentage of methylated CpGs of miR-124–1 was 37.5% in QSG-7701 and LO2, and 75% (12/16), 75% (12/16), 75% (12/16) and 56.25% (9/16) in MHCC-LM3, Huh7, MHCC-97L and HepG2, respectively, suggesting that overmethylation of miR-124-1 promoter downregulates its expression in HCC cell lines.

### MicroRNA-124-1 is downregulated and correlated with prognosis in hepatocellular carcinoma

**Figure 3:**
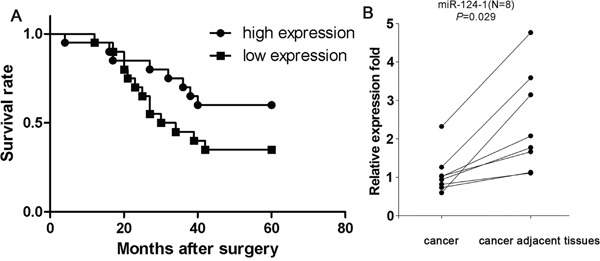
**A.** Kaplan-Meier survival analysis of HCC patients grouped by miR-124-1 expression level. We found that miR-124-1 expression was not significantly associated with overall survival in patients with HCC (P = 0.117). **B.** Comparison of miR-124-1 expression in 8 paired HCC tissues and adjacent non-cancer tissues by real-time PCR. The statistical significance of differences between cancer tissues and adjacent non-cancer tissues was calculated using Student's t-test. (P = 0.029).

Assessment of miR-124-1 expression in eight paired hepatocellular carcinoma samples by real-time PCR showed a significant downregulation of miR-124-1 expression in cancer tissues compared with the matched non-cancer tissues (P = 0.024; Figure 3B). To examine the possible correlation of miR-124-1 expression with clinical features or the prognosis of HCC patients, miR-124-1 expression levels were detected by real-time PCR in 40 formalin-fixed paraffin-embedded HCC tissue samples. Kaplan-Meier analysis revealed that the median survival time of patients with low miR-124 expression levels was 37.1 months, whereas the median survival time of patients with high miR-124-1 expression levels was 46.5 months (log-rank = 2.530, P = 0.1117, Figure 3A), indicating that miR-124-1 expression level was not significantly associated with overall survival in HCC patients. We further analyzed the correlation between miR-124-1 expression levels and pathological and clinical data, including pathological classification, TNM grade, pathologic stage, sex, Hepatitis B and Hepatitis C infection status (Table 1). The results showed that HCC patients with clinical stage I and II tended to show high miR-124-1 expression in cancer tissues compared to those with clinical stage IIIA (P = 0.048). Tumors penetrating the submucosa and muscularis propria (T1 and T2) had higher levels of miR-124-1 than tumors invading through the muscularis propria and invading other organs (T3 and T4) (P = 0.048). Meanwhile, we found that miR-124-1 level in HCC with Hepatitis C infection are lower than without Hepatitis C infection (P = 0.01). MiR-124-1 expression was not significantly correlated with sex, pathological grade and Hepatitis B infection status (P > 0.05). These data indicate that miR-124-1 expression could be a useful marker for the diagnosis of patients with HCC.

**Table 1:** Correlation between microRNA-124-1 expression levels and clinicopathological factors in hepatocellular carcinoma tissues (N=40)

	Number of patients	miR-124-1 expression*		p-value
		Low expression n=20	High expression n=20	
Gender				
Male	32	15	17	0.347
Female	8	5	3	
Histopathological grade				
G1	3	1	2	0.684
G2	20	11	9	
G3	16	7	9	
TNM grade				
T1, 2	14	4	10	0.048**
T3, 4	26	16	10	
Pathologic stage				
I~II	14	4	10	0.048**
IIIA	26	16	10	
Hepatitis B status				
Negative	11	6	5	0.716
Positive	29	19	10	
Hepatitis C status				
Negative	6	1	5	0.01**
Positive	34	26	8	

Abbreviations: HCC, hepatocellular carcinoma.

Grades 1–3 (or I–III) in Pathology Diagnosis are equivalent to well-differentiated, moderately-differentiated or poorly differentiated, respectively, under the microscope.

Grade 1 or well-differentiated: cells appear normal and are not growing rapidly.

Grade 2 or moderately-differentiated: cells appear slightly different than normal.

Grade 3 or poorly differentiated: cells appear abnormal and tend to grow and spread more aggressively.

Grade 4 or undifferentiated: *(for certain tumors) features are not significantly distinguished to differentiate from undifferentiated cancers, which occur in other organs

TNM grade:

T - Primary tumor (Tx - Primary tumor cannot be assessed; T0 - No evidence of primary tumor; Tis - Carcinoma in situ; intraepithelial or invasion of lamina propria; T1 - Tumor invades submucosa; T2 - Tumor invades muscularis propria; T3 - Tumor invades through muscularis propria into subserosa or into non-peritonealized pericolic or perirectal tissues; T4 - Tumor directly invades other organs or structures and/or perforate visceral peritoneum)

N - Regional lymph nodes (Nx - Regional lymph nodes cannot be assessed; N0 - No regional lymph node metastasis; N1 - Metastasis in 1 to 3 regional lymph nodes; N2 - Metastasis in 4 or more regional lymph nodes)

M - Distant metastasis (Mx - Distant metastasis cannot be assessed; M0 - No distant metastasis; M1 - Distant metastasis)

* For miR-124-1 expression, median values were used as the cut-off point for definition of subgroups (low expression and high expression groups).

** Compared with the independent sample t test; the other P-values were obtained using Fisher's exact test.

### MicroRNA-124-1 downregulates CASC3 expression by targeting its 3′UTR

**Figure 4:**
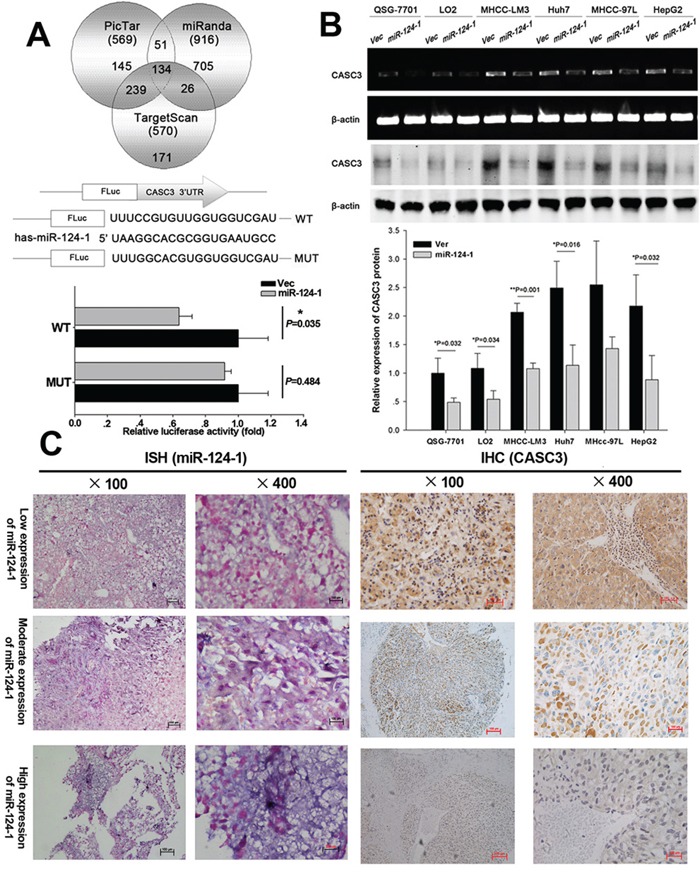
MicroRNA-124-1 downregulates CASC3 expression by directly targeting its 3′-UTR **A.** Putative miR-124-1 binding sequence in the 3′-UTR of CASC3 mRNA. A human CASC3 3′-UTR fragment containing the wild-type or mutant miR-124-1 binding sequence was cloned downstream of the luciferase reporter gene. HEK293 cells were cotransfected with miR-124 or a control vector and a luciferase reporter construct containing the wild-type or mutant CASC3 3′-UTR. Luciferase activity was assayed 48 h after transfection. Firefly luciferase activity of each sample was normalized by Renilla luciferase activity. Data were normalized to the luciferase activity detected in cells transfected with the control vector, and the luciferase activity of the control vector was not significant. **B.** Effects of miR-124-1 overexpression on endogenous CASC3 expression as measured by real-time PCR and western blotting. β-actin served as the internal control. **C.** miR-124-1 and CASC3 protein expression were examined by in situ hybridization and immunohistochemistry, respectively.

To further explore the downstream effects of miR-124-1, potential targets of miR-124-1 were identified using the TargetScan, Pictar and miRanda databases. The results showed that 134 genes may be regulated by miR-124-1 (potential targets of miR-124-1 were listed in supplementary 2). Among them, CSAC3 was selected for further experimental validation because of its frequent overexpression in tumor tissues and well-known importance in tumorigenesis. To determine whether miR-124-1 regulates CASC3 through direct binding to its 3′-UTR, wild-type or mutant fragments of the CASC3 3′UTR were inserted immediately downstream of the luciferase reporter gene. For luciferase assays, miR-124-1 mimics were co-transfected with the different luciferase 3′-UTR constructs into HEK293 cells. miR-124-1 decreased the relative luciferase activities in the presence of the wild-type 3′-UTR, whereas the decrease in luciferase activity was attenuated in the mutant constructs of CASC3 (Figure 4A). Consistent with these results, CASC3 mRNA and protein expression was downregulated in normal liver and HCC cell lines ectopically expressing miR-124-1 (Figure 4B). Furthermore, miR-124-1 was downregulated and CASC3 was overexpressed in HCC tissues (Figure 4C). These results suggest that miR-124-1 downregulates CASC3 expression by directly targeting its 3′-UTR.

### miR-124-1 functions to inhibit HCC cell proliferation by targeting CASC3

**Figure 5:**
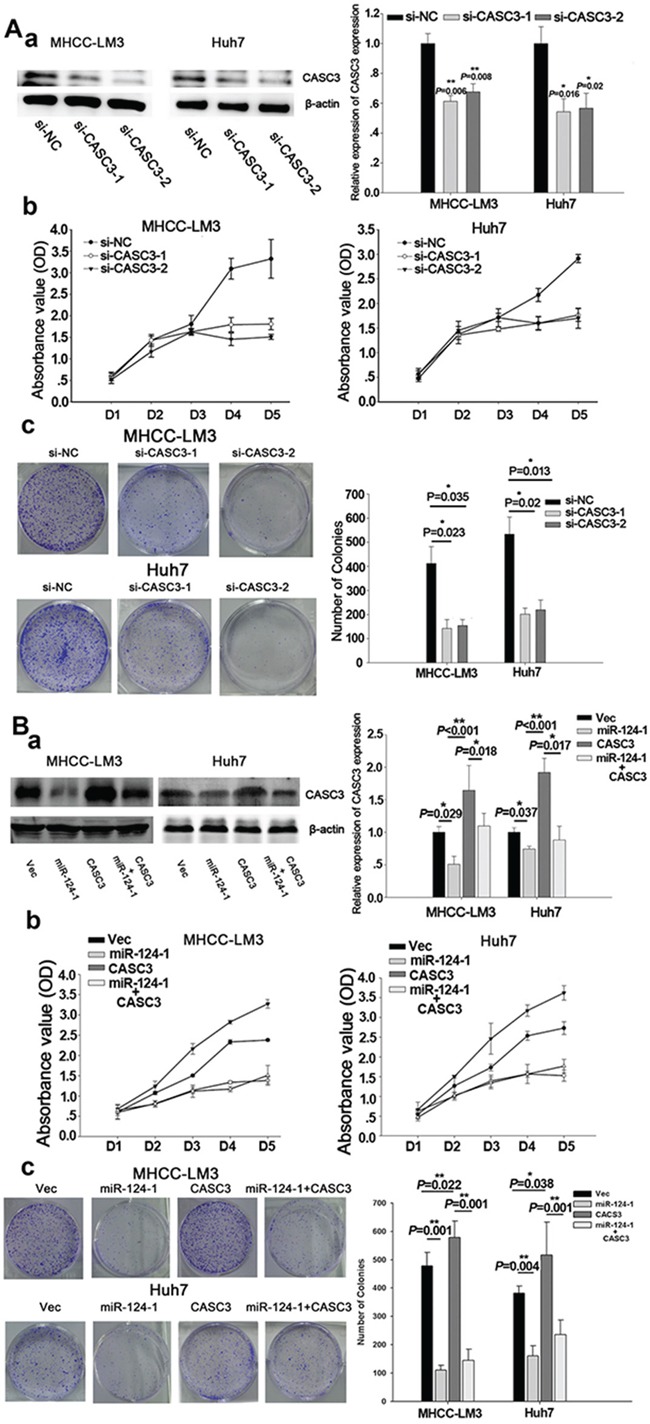
CASC3 mediates the tumor-suppressive function of miR-124-1 **A.** MHCC-LM3 and Huh7 cells were transfected by siRNA for CASC3. **B.** MHCC-LM3 and Huh7 transfectants stably expressing CASC3, miR-124-1 and control vector were generated using a lentiviral infection system. CASC3 protein expression levels were confirmed by western blotting (A-a, B-a). HCC cell growth was measured by CCK-8 (A-b, B-b) and colony formation assays (A-c, B-c). The results are presented as the mean±sd of values obtained in three independent experiments. Statistical significance was calculated using the Student's t-test. * P<0.05.

To confirm the effects of CACS3 on tumor cell growth, CASC3 expression was knocked down by siRNA in MHCC-LM3 and Huh7 cells (Figure 5A-a), which showed that silencing of CASC3 significantly inhibited HCC cell proliferation (Figure 5A-b). Colony formation assays showed that silencing of CACS3 inhibited colony formation in the two cell lines (Figure 5A-c). To further evaluate the miR-124-1 inhibition of HCC cell growth and proliferation mediated by its target CASC3, we established stably overexpressing miR-124-1 MHCC-LM3 and Huh7 transfectants by infecting the cells with lentivirus encoding CASC3 (Figure 5B-a). We found that overexpression of CASC3 significantly increased HCC cell proliferation and colony formation. *In vitro* cell proliferation assays revealed that overexpression of miR-124-1 significantly inhibited HCC cell proliferation. Colony formation assays confirmed that cells with enhanced miR-124 expression formed fewer and smaller colonies than control cells. CASC3 reversed the effects of miR-124-1 on HCC cells (Figure 5B-b, c), suggesting that CASC3 is a functional target of miR-124-1.

### MicroRNA-124-1 suppresses the tumorigenesis of HCC cells *in vivo*

**Figure 6:**
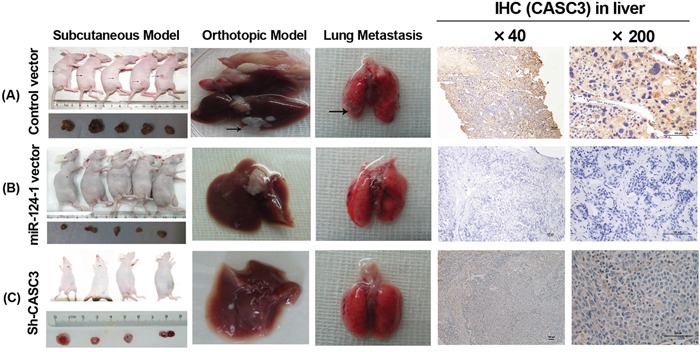
The effect of miR-124-1 on tumor formation in a nude mice xenograft model Nude mice were injected subcutaneously in opposite flanks with 5×10^6^ control lentiviral vector-infected cells and miR-124-1 vector-infected cells. After 2 weeks, the mice were sacrificed when the tumors reached 1.0 cm in diameter and the subcutaneous tumors were cut into 1.0 mm^3^ sections, which were then inserted into the livers of another 10 nude mice. The mice were followed for 30 days and then killed by cervical dislocation. Livers and lungs were resected and imaged with a high-definition digital camera. Each group was composed of 5 mice. The weight of the tumors in the two groups was compared using the Student's t-test. **A.** control lentiviral vector groups; **B.** miR-124-1 vector groups; **C.** Sh-CASC3 groups.

To further examine the effect of miR-124-1 on the inhibition of tumor growth *in vivo*, nude mice were subcutaneously injected with MHCC-LM3-Ver, MHCC-LM3-miR-124-1 and MHCC-LM3-Sh-CASC3 cells; all groups successfully formed tumors 15 days post inoculation (Figure 6). The tumor volumes of the MHCC-LM3-miR-124-1 and MHCC-LM3-Sh-CASC3 induced tumors were significantly reduced (Figure 6A). Next, all transfected cells were orthotopically transplanted into the livers of mice. Ten weeks after injection, the mice were euthanized, and the livers and lungs were harvested. All groups formed tumors, but miR-124-1 overexpression or CASC3 inhibition significantly slowed the growth of liver tumors (Figure 6B and 6C). Interestingly, 60% of all animals (3/5) in the control vector group had developed lung metastasis (Figure 6A). These findings indicate that miR-124-1 effectively suppresses the tumorigenesis and metastasis of HCC cells though the inhibition of the expression of CASC3 *in vivo*. To explore the expression of CASC3, tissues derived from orthotropic liver cancers were immunostained with an antibody against CASC3. Consistent with the previous results, CASC3 expression was significantly lower than the control groups. Taken together, these findings indicate that miR-124-1 effectively suppresses the tumorigenesis and metastasis of HCC cells though the inhibition of the expression of CASC3 *in vivo*.

### MicroRNA-124-1 and CASC3 affect HCC cell growth through the p38/ERK/JNK pathway

**Figure 7:**
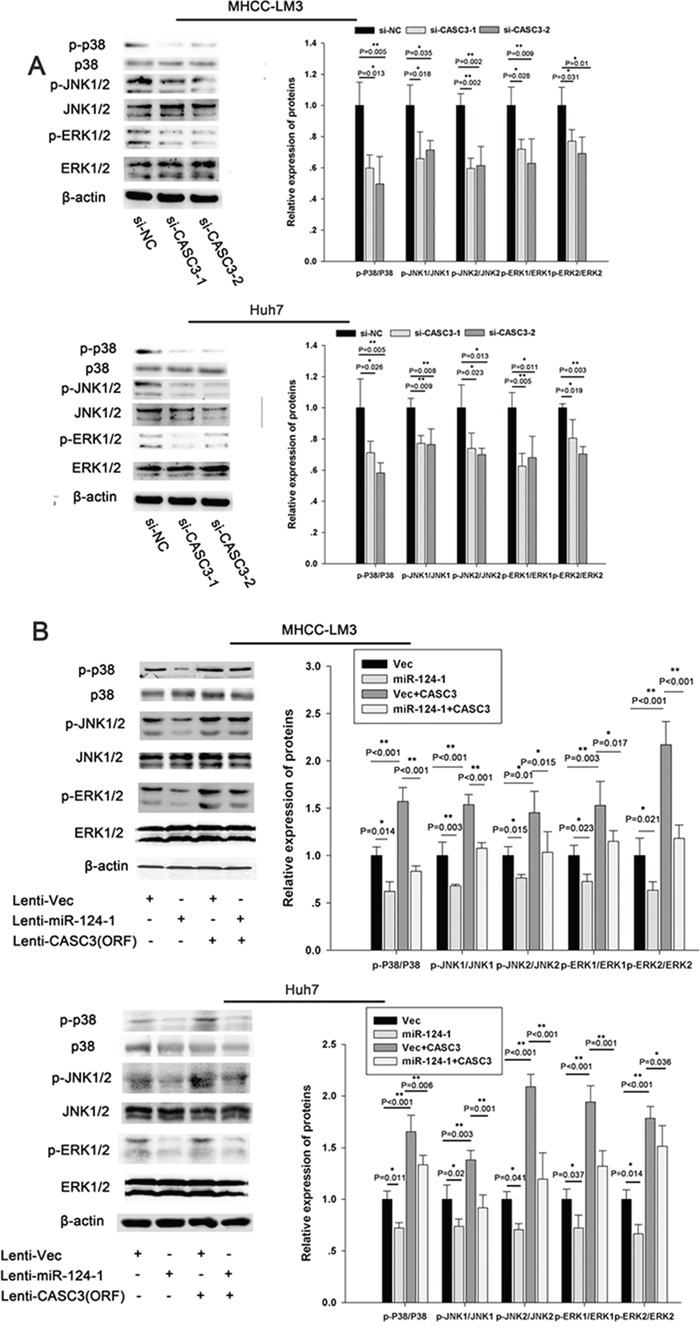
miR-124-1 inhibits tumorigenesis via the p38-Akt-JNK pathway **A, B.** The activity of the JNK pathway was evaluated in MHCC-LM3 (A) and Huh7 (B) cells transfected with si-CASC3. The activity of the JNK pathway in HCC cells infected with a control lentiviral vector or an miR-124-1expression lentiviral vector was assessed after transduction with a lentiviral vector encoding CASC3 (open reading frame without the 3′-UTR).

**Figure 8:**
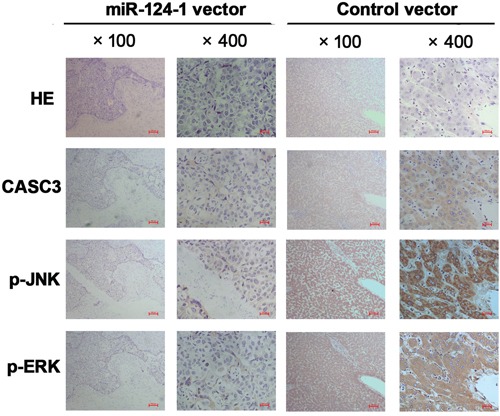
Immunohistochemistry was performed on HCC tissues from orthotopical implantation models of miR-124-1 vector and control vector transfected MHCC-LM3 cells for CASC3, p-JNK, and p-ERK The photomicrographs were obtained at ×100, and ×400 magnification.

To investigate whether miR-124-1 is involved in HCC progression through regulation of the p38 MAPK/Akt pathway, we used cells with low miR-124-1 expression (MHCC-LM3 and Huh7) and analyzed the expression of components of the p38 MAPK/Akt pathway. We found that inhibition of CASC3 expression significantly downregulated the expression of p-p38, p-ERK, and p-JNK in HCC cell lines (Figure 7A). We also found that miR-124-1 overexpression decreased the activity of this pathway, whereas ectopic expression of CASC3 blocked the miR-124-1 induced inactivation of the P38 MAPK pathway (Figure 7B). Moreover, the expression levels of these proteins in HCC tissues from orthotopical implantation models of miR-124-1 transfected MHCC-LM3 cells were markedly increased compared with the controls (Figure 8). These results indicate that miR-124-1 suppresses tumorigenesis by inhibiting CASC3 expression, which affects the activity of the p38/MAPK/Akt signaling pathways.

## DISCUSSION

The development of HCC is a multistep process driven by genetic alterations that activate oncogenes or inactivate tumor suppressor genes. These factors constitute an important limitation to the design of effective therapeutic strategies for the treatment of HCC. Recent studies have shown that miRNAs play a fundamental role in the development of HCC. Many studies have shown that the normal expression of miRNAs can be affected by chromosomal translocation, amplification, or deletion.

In the present study, we reviewed the literature and found that loss of chromosomes 8p23.3-21.3 is the most common chromosomal alteration in HCC. This could lead to the downregulation of the expression of miRNAs encoded in this chromosome fragment. Further experiments showed that miR-124-1 (located in 8p23.1) is a tumor suppressor in HCC. In fact, miR-124-1 has been confirmed to be involved in several human solid cancers [41–43], but its role in HCC has not yet been reported. In the present study we showed that miR-124-1 expression was reduced in HCC tissues, and its downregulation was significantly associated with certain clinical characteristics, such as TNM grade or pathologic stage. Although our data show that miR-124-1 expression is not correlated with the survival of patients (P = 0.117), this could be attributed to the small number of patient samples analyzed. Future experiments will include a larger number of samples to investigate the correlation between miR-124-1 expression and patient outcome in HCC.

We also found that hypermethylation of the miR-124-1 promoter was associated with the downregulation of its expression in HCC cell lines, and demethylation treatment with 5-aza-2-deoxycytidine recovered the expression levels of miR-124-1. These data indicated that a major mechanism for the downregulation of miR-124-1 is hypermethylation.

We identified CASC3 as a target of miR-124-1 associated with its effects on tumorigenesis in HCC. CASC3, also known as MLN51, was first identified in breast cancer cells [44], and it was reported to be associated with several diseases including malignant tumors [45, 46]. In the current study, we observed that CASC3 overexpression promoted HCC cell proliferation and colony formation, and these effects were confirmed *in vivo* in a xenograft tumor model. MiR-124-1 restoration not only significantly decreased the expression of CASC3, but also attenuated the tumor-promoting activity of HCC cells *in vitro*.

We know that CASC3 is an EJC component and have role in the splicing regulation of long intron-containing genes. In recent studies, it has been proven that it controls RAS/MAPK signaling in Drosophila [47]. Indeed, Alternative splicing factors have also been identified as a target of multiple MAP kinases, such as ERKs, JNKs and p38 MAPK [48]. Interesting, CASC3 (called Barentsz in Drosophila) depletion does not impact the splicing of MAPK or of other pre-mRNAs in previous studies [47, 49]. While in another study, CASC3 acts independently of EIF2α phosphorylation through subsequent translational control in HepG2 cells. And the process is associated with MAPK signaling pathway [50]. CASC3 is also considered to a stress-induced agent which incorporates in mRNP complex generated by splicing and increases some cell features in the stress response including in oncogenesis [51, 52]. Alternative splicing can affect the activity of signaling effectors contributing to their role in cancer development and progression [53]. We suspected that it may not affect alternative splicing and expression of MAPK, but the influence of phosphorylation through stress-induced changes. So we detected certain proteins phosphorylation levels of MAPK signaling. Similarly, our results indicated that miR-124-1 mediated CASC3 silencing might provide the critical link between p38 MAPK, ERK and JNK signaling.

The levels of the activated forms of p38 MAPK, ERK and JNK were significantly increased in correlation with the upregulation of CASC3 expression in HCC cell lines, whereas overexpression miR-124-1 reversed this effect. Furthermore, we performed IHC on serial sections of HCC tissues and showed that the expression of p-MKK4, p-JNK and p-c-Jun correlated inversely with miR-124-1 expression in HCC. Thus, our current study suggests that activation of the p38 MAPK, ERK and JNK pathways by CASC3 plays a key role in miR-124-1 silencing-induced tumorigenesis in HCC.

In summary, the present study showed that HCC progression may be associated with epigenetic regulation through miR-124-1 methylation and that downregulation of miR-124-1 may be involved in the pathogenesis of HCC. In addition, our data suggest that the effect of miR-124-1 on the development of HCC is mediated by its target CASC3 and the p38 MAPK-ERK-JNK pathway. Our findings may provide useful clues to improve our understanding of the processes that lead to cancer development.

## MATERIALS AND METHODS

### Cell culture

Two benign liver cell lines (LO2 and QSG-7701) and four HCC cell lines (MHCC-LM3, Huh7, MHCC-97L and HepG2) were purchased from the Chinese Academy of Sciences. The cell lines were maintained in high glucose DMEM (Thermo Fisher) supplemented with 10% FBS in a humidified 5% CO2 atmosphere at 37°C.

### Patients and clinical specimens

HCC samples were collected from 40 patients who underwent surgical resection between January 2005 and December 2010 at Shanghai Tenth People's Hospital. All of the patients underwent macroscopically curative resection. None of the patients had received neoadjuvant radio or chemotherapy. Fresh HCC tissues and their adjacent normal samples were obtained for analysis.

### Methylation assay

Genomic DNA was isolated from cell lines. Bisulfite modification of DNA (1.0 mg) was performed using an EZ DNA methylation-direct kit (Zymo Research, Irvine, CA, USA). In cell lines, the methylation of 5′-CpG island regions was detected by methylation specific PCR (MSP) using primers specific for either methylated or unmethylated DNA. The COBRA assay was performed as previously described [11]. The primers used for MSP and COBRA are listed in Supplementary Table 1.

### RNA isolation and quantitative real-time PCR

Total RNA was extracted using the TRIzol Reagent (Invitrogen, San Diego, CA, USA). First-strand cDNA was synthesized from 2 μg of total RNA. Amplification and detection were tested using the ABIPRISM 7900 Sequence Detection System (Applied Biosystems) starting with 1 ll cDNA and SYBR Green Real-time PCR Master Mix (Takara). The comparative cycle threshold (Ct) method was used to analyze the relative expressions of specific mRNAs and miR-124-1. The primer sequences are listed in Supplementary Table 1.

### Plasmid construction and luciferase reporter assay

A partial human pri-miR-124-1 gene was subcloned into the lentiviral expression vector pWPI-GFP. The coding sequence of human CASC3 was amplified and cloned into another lentiviral expression vector, pCDHCMV-MCS-EF1-Puro, to generate pCDH-CASC3. Biologically active short hairpin RNAs were generated using the lentiviral expression vector pLKO.1-puro. The short hairpin RNA sequences for human CASC3 were 5′-CCGG GCCCATGTCTTCTGCTGTTCTTTCAAGAGAAGAACAGCAGAAGACATGGGCTTTTTGGAA-3′ and 5′-CACCGGATTATGAAAGTGCAGAAGACGAATCT TCTGCACTTTCATAATCC-3′. pLKO.1-shLuc, which targeted the luciferase gene, was used as a control for RNA interference. The full length 3′UTR of CASC3 (Genbank Accession: NM_007359.4) was produced by annealing the sense strand to form 5′ Xhol and 3′ Not I sites, which were used for ligation into psiCHECK-2 dual-luciferase reporter plasmid with the stop codon including the Renilla luciferase gene. The binding sequences for miR-124-1 in the 3′UTR of CASC3 were mutated at positions 1520–1525 from CCGUGU to GGCACG. For luciferase reporter assay, cells were transfected for 48 h, harvested and lysed with passive lysis buffer (Promega). Luciferase activity was measured using a dual luciferase reporter assay (Promega). Luciferase activity was normalized to Renilla luciferase activity.

### RNA interference (RNAi) of CASC3 and transfection assay

HCC cell lines (MHCC-LM3 and Huh7) were transfectd with 50nM CASC3 siRNA or control siRNA using LipofectamineTM 2000 reagent (Invitrogen). After 48h of transfection, the cells were processed for the quantification of proteins. Second siRNA sequences have been designed to discard off-targets effects. CASC3 siRNA1: 5‘-AUUAGCUUCUGAUUUCAGCUC-3‘, CASC3 siRNA2: 5‘-AAUCUCAUGCUUAACAGUCUC-3‘.

### Cell proliferation assays using the CCK8 kit

Cell proliferation was measured using the CCK-8 kit (cell counting kit-8) (Dojido, Japan) according to the manufacturer's instructions. Briefly, 5–10×10^3^ cells were seeded into 96-well culture plates. At the indicated endpoint, 20 μl CCK-8 (5 g/L) was added for further 4 h. Absorbance at 450 nm was measured using a Victor3 microplate reader (Perkin-Elmer, Waltham, MA, USA).

### Colony formation assays

Colony formation assays were performed as described previously [12]. Exponentially growing cells were used to generate single-cell suspensions (1 × 10^5^ cells/mL). Aliquots of 0.2 ml of cell suspension (containing 2000 viable cells) and 4 ml of culture medium were added to each well of a 6-well culture plate, which was incubated at 37°C for 2 weeks, washed twice with warm PBS, and stained with Giemsa solution. The number of colonies was counted under a microscope (40×).

### Assessment of tumorigenicity *in vivo*

Tumorigenicity *in vivo* was analyzed as described in our previous report [13, 14].

### Western blot analysis

Western blot analysis was performed as described in our previous report [13, 14]. These primary antibodies are as followed:β-actin (ab8227) (1:3,000), CASC3 (ab90651), p38 (ab31828), phospho p38 (ab47363), ERK1/2 (ab17942), phospho ERK1/2 (ab50011), JNK1/2 (ab112501), and phospho JNK1/2 (ab131499) (1:1,000; ABcam, Cambridge, MA, USA).

### Immunohistochemical analysis

Immunohistochemical analysis was performed as described in our previous report [13, 14]. CASC3 staining was cytoplasmic, and P-JNK and P-ERK staining were performed in the nucleus and cytoplasm in tumor tissues.

### In situ hybridization

In situ hybridization was performed to detect miR-124-1 expression in HCC tissues. In brief, sections were deparaffinized, rehydrated, digested and refixed in 4% paraformaldehyde. Sections were then reconstituted with hybridization solution and incubated at 68°C for 20 h with a digoxigenin-labeled probe (Exiqon, Vedbaek, Denmark) diluted to 250 nM in hybridization buffer at 50°C for 2 h. Stringent washes were performed with 5×SSC, 1×SSC and 0.2×SSC buffers at 50°C over 33 min. Sections were incubated in DIG blocking reagent (Roche) in maleic acid buffer containing 2% sheep serum at 30°C for 15 min, alkaline phosphatase-conjugated anti-digoxigenin (diluted 1:500 in blocking reagent, Roche) at room temperature for 60 min. Enzymatic development was performed using 4-nitro-blue tetrazolium and 5-brom-4-chloro-3-Indolyl phosphate substrate (Roche), which forms a dark-blue NBT-formazan precipitate at 30°C for 120 min, followed by nuclear fast counterstain for 5 min. The slides were then dismantled in water, dehydrated in alcohol solutions and mounted with eukitt mounting medium (VWR, Herlev, Denmark). Scrambled probe was used as a control. Signals were visually quantified using a quick score system from 0 to 5, combining intensity of signal and percentage of positive cells (signal: 0 = no signal, 1 = weak signal, 2 = intermediate signal, 3 = strong signal; percentage: 0 = 0%, 1 = <30%, 2 = >30%). Tissue sections were blindly examined by a second individual and this yielded a good agreement with the initial quantifications.

### Statistical analysis

Data were expressed as mean ± SE. Statistical analysis of variance and Student's t-tests were used to determine the statistical significance of differences between samples. RT-PCR, clone formation, CCK-8 analysis and *in vitro* invasion assays were examined using one-way ANOVA. The χ2 and Fisher's exact test were used to analyze the association between miR-124-1 expression and pathological parameters. Values of P<0.05 was considered statistically significant.

### Ethics statement

All the methods were carried out in accordance with the approved guidelines. For Patients and clinical specimens, the informed consent was obtained from all patients, the entire experimental procedures were carried out under the guidance of the Animal Care and Use Committee of Shanghai Tongji University.

## SUPPLEMENTARY TABLES




