# Supplementary material for: Methylation-regulated miR-124-1 suppresses tumorigenesis in hepatocellular carcinoma by targeting CASC3
Source: Oncotarget. 2016 Mar 22;7(18):26027–41. doi: 10.18632/oncotarget.8266 (PMC5041962; doi:10.18632/oncotarget.8266)
Supplement: Supplementary file 1 [file oncotarget-07-26027-s001.pdf]

SUPPLEMENTARY TABLES

Supplementary Table 1: Primers for MSP and Real-time stem-loop RT- PCR

Gene	MSP/USP	Primer Sequence		Length
		**Forward (5'-3')**	**Reverse (5'-3')**	
	MSP	AGAGGATTGTAGTAGGCGAGTTTC	AACGAAAAACAAAAAAAACAACG	190
miR-124-1	USP	GAGGATTGTAGTAGGTGAGTTTTGG	ACAAAAAACAAAAAAAACAACAAA	188
	COBRA	AGTAGATTTATTGTTTTTGGTTGGG	CCACAAAAACAAATAAAAAACAATC	241
Gene		Primer Sequence		Length
miR-124-1	RT primer (5'-3')	GTCGTATCCAGTGCAGGGTCCGAGGTATTCGCACTGGATACGACATCAAG		
	Forward (5'-3')	CGCCGTGTTCACAGCGGAC		87
	Reverse (5'-3')	GTGCAGGGTCCGAGGT		
U6	RT primer (5'-3')	CGTTCACGAATTTGCGTGTCAT		
	For ward (5'-3')	GCTTCGGCAGCACATATACTAAAAT		89
	Reverse (5'-3')	CGCTTCACGAATTTGCGTGTCAT		
CASC3	Forward (5'-3')	ACCTCGGAAAGGGCTCTTCTT		105
	Reverse (5'-3')	CGACCCTCATCCTTCCATAGC		
β-actin	Forward (5'-3')	CTGGAACGGTGAAGGTGACA		140
	Reverse (5'-3')	AAGGGACTTCCTGTAACAATGCA		

Supplementary Table 2. Literatures concerning analysis of the genomic instability of HCC

See Supplementary File 1

Supplementary Table 3: The most common chromosomal losses in HCC

Chromosome	Loss numbers	Total numbers	Incidence rate
1p	325	791	0.410872
4q	820	1709	0.479813
6q	157	439	0.357631
8q	886	1840	0.481522
13q	349	917	0.380589
16q	657	1412	0.465297
17q	494	1078	0.458256
19p	23	62	0.370968
