# Supplementary material for: Methylation-regulated miR-124-1 suppresses tumorigenesis in hepatocellular carcinoma by targeting CASC3
Source: Oncotarget. 2016 Mar 22;7(18):26027–41. doi: 10.18632/oncotarget.8266 (PMC5041962; doi:10.18632/oncotarget.8266)
Supplement: Supplementary file 2 [file oncotarget-07-26027-s002.doc]

Supplementary Table 2. Literatures concerning analysis of the genomic instability of HCC

| **References** | **Breakpoints** | |
| --- | --- | --- |
|  | Loss/Gain | Location (number or ration) |
| **13** | Gain | 1q (19/32), 8q(15/32), 6p(12/32), 17p(7/32) |
|  | Loss | 1p(7/32), 4q(8/32), 6q(7/32), 8p(20/32), 13q(9/32), 14q(7/32), 16q(5/32), 17q(7/32) |
| **14** | Gain | 1q(4/12), 3q(4/12), 6p(2/12), 7p(1/12), 8q(4/12) |
|  | Loss | 1p(5/12), 8p(4/12), 10q(1/12), 14p(2/12), 17p(3/12), 19p(2/12) |
| **15** | Gain | 1q(33/50), 8q(24/50), 20q(10/50) |
|  | Loss | 16q(35/50), 17p(26/50), 19p(21/50), 4q(20/50), 1p(18/50), 8p(15/50), 22q(14/50) |
| **16** | Gain | —— |
|  | Loss | 1p36(15/24), 13p14(7/24), 17p13(9/24), 4q28(11/24) |
| **17** | Gain | 1q, 8q, 17q |
|  | Loss | 1p(4/7), 4q(4/7), 6q(1/7), 8p(5/7), 13q(4/7), 16q(4/7), 17p(4/7), Xq(3/7) |
| **18** | Gain | 1q, 8q, 17q |
|  | Loss | 1p(4/7), 4q(4/7), 6q(1/7), 8p(5/7), 13q(4/7), 16q(4/7), 17p(4/7), Xq(3/7) |
| **19** | Gain | —— |
|  | Loss | 1p(76%), 4q(75%), 13q(73%), 17p(73%), 9p(72%), 8p(67%), 1q(66%), 16q(65%), 3p(62%), 2q(58%), 6q(56%) |
| **20** | Gain | —— |
|  | Loss | 1p(39/56), 4q(40/56), 8p(37/56), 16q(37/56), 17p(36/56) |
| **21** | Gain | 1q(38%), 8q(50%), 17q(30%) |
|  | Loss | 4q(36%), 8p(29%), 16q(28%) |
| **22** | Gain | 8q(25/87), 17q(20/87) |
|  | Loss | 4q(14/87), 8p(69/87), 13q(39/87),14q(14/87), 17p(32/87) |
| **23** | Gain | 1q(77%), 6p(48%), 8q(45%), 17q(32%), 20q(26%),11q(23%) |
|  | Loss | 13q(64%), 4q(48%), 8p(39%), 16q(39%), 16p(35%), 9p(26%) |
| **24** | Gain | —— |
|  | Loss | 8p(8/19), 11p(6/19) |
| **25** | Gain | 17q(65%), 9q(55%) |
|  | Loss | 16q(52%), 4q(40-51%), 17p(49%), 13q(46%), 8p(41-45%) |
| **26** | Gain | 8q(83%), 1q(73%), Xq(50%), 7q(40%), 5p(38%) |
|  | Loss | 17p(60%), 16p(46%), 8p(44%), 4q(40%) |
| **27** | Gain | 1q (78%), 8q (66%) |
|  | Loss | 17p(51%), 16q(46%), 13q(37%), 4q(32%), 8p (29%), 10q(17%) |
| **28** | Gain | 8q (30%), 1q (20%), 6p (20%), 17q (18%) |
|  | Loss | 16q (43%), 17p (20%), 13q (20%), 4q (15%), 8p (15%) |
| **29** | Gain | 8q(60%), 1q(58%), 6p(33%), 17q(33%) |
|  | Loss | 4q(70%), 8p(65%), 16q(54%), 17p(51%), 13q(37%), 6q(37%), 1p(30%) |
| **30** | Gain | 1q(10/10), 8q(6/10), 5p(3/10) |
|  | Loss | 4q(7/10), 8p(3/10), 17p(5/10), 1p(6/10) |
| **31** | Gain | 1q(46%), 6p(20%), 8q(31%), 17q(43%) |
|  | Loss | 1p(37%), 4q(48%), 5q(35%), 6q(23%), 8p(28%), 13q(20%), 16q (33%),17p(37%) |
| **32** | Gain | 1q(46%), 6p(20%), 8q(41%), 11q(27%), 17q(37%) |
|  | Loss | 1p(24%), 4q(39%), 6q(41%), 8p(44%), 9p(24%), 11q(24%), 12q(22%), 13q(39%) |
| **33** | Gain | 1q (48/67), 8q (32/67), 17q (20/67), 20q (25/67) |
|  | Loss | 4q (29/67), 8p (25/67), 13q (25/67), 16q (20/67) |
| **34** | Gain | —— |
|  | Loss | 4q (75%), 8p (70%), 16q (65%). |
| **35** | Gain | 8q(69%), 1q(46%), 17q(46%), 12q(42%), 20q(31%), 5p(27%), 6q(27%), Xq(27%) |
|  | Loss | 8p(58%), 16q(54%), 4q(42%), 13q(39%), 1p(35%), 4p(35%), 16p(35%), 18q(35%), 14q(31%), 17p(31%), 9p(27%), 9q(27%) |
| **36** | Gain | 1q(71%), 5q(21%), 6p(42%), 7q(57%), 8q(64%), 9q(64%), 12q(36%), 13q(29%), 16p(57%), 17q(92%), 20q(64%) |
|  | Loss | 1p(29%), 4q(79%), 6q(29%), 8p(36%), 9p(36%), 10q(21%), 14q(43%), 18q(29%) |
| **37** | Gain | 1q (91.6%), 8q (58.3%) |
|  | Loss | 8p (54%) |
| **38** | Gain | 1q(86%), 8q(77%). |
|  | Loss | 4q(59%), 8p(77%), 16q(50%) |
